# Impact of Fermented Soy Beverages Containing Selected Vaginal Probiotics on the In Vitro Fecal Microbiota of Post-Menopausal Women

**DOI:** 10.3390/foods14061022

**Published:** 2025-03-17

**Authors:** Margherita D’Alessandro, Davide Gottardi, Silvia Arboleya, Guadalupe Monserrat Alvarado-Jasso, Carola Parolin, Beatrice Vitali, Rosalba Lanciotti, Miguel Gueimonde, Francesca Patrignani

**Affiliations:** 1 Interdepartmental Center for Industrial Agri-Food Research, University of Bologna, Via Quinto Bucci 336, 47521 Cesena, Italy; rosalba.lanciotti@unibo.it (R.L.); francesca.patrignani@unibo.it (F.P.); 2 Department of Agricultural and Food Sciences, University of Bologna, Piazza Gabriele Goidanich 60, 47521 Cesena, Italy; 3 Department of Microbiology and Biochemistry of Dairy Products, Dairy Research Institute IPLA-CSIC, C/Francisco Pintado Fe 26, 33011 Oviedo, Spain; silvia.arboleya@ipla.csic.es (S.A.); gpemonserratjasso@gmail.com (G.M.A.-J.); mgueimonde@ipla.csic.es (M.G.); 4 Department of Pharmacy and Biotechnology, University of Bologna, Via San Donato 19/2, 40127 Bologna, Italy; carola.parolin@unibo.it (C.P.); b.vitali@unibo.it (B.V.)

**Keywords:** probiotics, vaginal lactobacilli, soy beverages, gut microbiota, post-menopausal women, microbial dysbiosis

## Abstract

The gut microbiome of women can change after menopause, and during this phase women can also be more susceptible to vaginal dysbiosis. Recent studies have explored the probiotic potential of *Lactobacillus crispatus* BC4 and *Lactobacillus gasseri* BC9 against various pathogens and their use as co-starters in foods. However, their effects on the gut microbiota of post-menopausal women, who are more prone to dysbiosis, have not been examined. This study investigated the effects of predigested soy beverages (INFOGEST) containing BC4 and BC9 (encapsulated or not) on the composition and metabolic activity of the gut microbiota in post-menopausal women, using a fecal batch culture model. Parameters such as pH, gas, SCFAs, and microbiota composition (targeted qPCR and 16S rRNA gene sequencing) were assessed. The study, while highlighting a strong variability among donors, showed differences in gut microbiota response to the tested products. For instance, donor 2 showed a significant increase in bifidobacteria with BC4 + BC9 and E-BC9, while BC4 increased *Ruminococcaceae* in donors 1 and 3, and E-BC4 and E-BC9 enhanced *Akkermansia* in donor 1. BC4, E-BC4, E-BC9, and E-BC4 + BC9 significantly impacted metabolic activity, as measured by SCFAs, compared to other samples. However, no significant differences in gas production were observed.

## 1. Introduction

The human gut microbiota, conceived as a symbiotic microbial community that acts like an organ fully integrated into the host, represents a very complex ecosystem [1]. Its composition is considered relatively stable during adult life, but changes may occur due to sex- and age-related conditions, such as menopause and post-menopause [2]. According to Peters et al. [3] post-menopausal women show lower gut microbial diversity, and a changed composition compared to pre-menopausal women. Zhao et al. [4] also reported lower microbial diversity and differences in the composition of the gut microbiome of post-menopausal women compared to pre-menopausal ones. In addition, 25–50% of post-menopausal women experience multiple vulvovaginal symptoms [5]. Reduced estrogen secretion, during and after menopause, leads to a decrease in lactobacilli and an increase in intravaginal pH, which enables harmful microorganisms (such as *Escherichia coli*, *Candida*, *Enterobacter*, *Gardnerella* etc.) to colonize the vagina, leading to bacterial vaginosis (BV) and complicated vulvovaginal candidiasis (VVC) [6,7].

In this context, the use of oral probiotic therapies, including healthy vaginal lactobacilli as active functional microorganisms, represents a promising solution to maintain a healthy vaginal environment or to treat its dysbiosis [8,9,10,11,12,13]. Several studies [14,15,16,17] have reported that oral probiotic formulations, as well as vaginal instillation, have the potential to restore vaginal microbiome.

Therefore, continued research into new strains with functional properties and different origins is very important. Parolin et al. [12] isolated vaginal strains of *Lactobacillus crispatus* and *Lactobacillus gasseri* with activity against several genital pathogens, including *Candida* [12], *Chlamydia trachomatis* [11], *Neisseria gonorrhoeae* [9], Group B *Streptococcus* [18], and HIV1 [19]. Although these strains could be exploited as oral formulation, their use in food as functional adjuncts could represent a daily nutritional and dietary strategy to prevent vaginal disorders. In fact, the idea of administering probiotics through foods that consumers appreciate could not only promote their consumption, but also allow a more constant intake compared to pharmaceutical preparations. For this reason, Siroli et al. [20] evaluated the technological properties of 17 vaginal lactobacilli, previously mentioned and isolated by Parolin et al. [12], for their potential application in dairy products, while Patrignani et al. [21] tested the behavior of one of these strains (namely *L. crispatus* BC4) added in cheese, using the SHIME^®^ system (ProDigest, Ghent University, Ghent, Belgium). The strain was able to survive simulated stomach and small intestine conditions, reaching the gut with a slight reduction in living cells. This aspect is important because once oral vaginal strains have colonized the intestine, they can translocate from the colon to the vagina due to the proximity of the two organs, having a positive effect on the vaginal habitat, as demonstrated by several investigations [14,17,22,23].

Therefore, the selection of food delivery system is very important because it can affect the survival of probiotics. Usually, fermented dairy products, mainly of bovine origin, are used. However, an increasing number of consumers are reluctant toward dairy products, especially for daily consumption. Thus, there is a growing demand for dairy-free alternatives such as plant-based beverages, more specifically soy-based ones, where probiotic lactic acid bacteria can remain at a very high level during the product’s entire shelf-life [24]. A recent study performed by D’Alessandro et al. [25] reported the suitability of selected functional vaginal probiotics (*L. crispatus* BC4 and *L. gasseri* BC9), encapsulated or not, as food adjuncts for the development of fermented soy beverages. However, the authors only highlighted the ability of these strains to achieve high cell counts throughout the product’s entire shelf life, providing a distinctive, pleasant flavor and aroma, without evaluating their effect on the gut microbiota of women potentially experiencing dysbiosis.

Based on these considerations, the aim of this work was to investigate the impact of fermented soy beverages, formulated with encapsulated or non-encapsulated *L. crispatus* BC4 and *L. gasseri* BC9, upon INFOGEST on the metabolic activity and composition of post-menopausal women microbiota. A batch colon incubation, prepared with feces from post-menopausal women donors, was used. pH, gas production, short-chain fatty acids (SCFAs), targeted quantification of lactobacilli, bifidobacteria and *Enterobacteriaceae*, and fecal microbiota composition using 16S rRNA gene sequencing were evaluated.

## 2. Materials and Methods

### 2.1. Bacterial Strains

The vaginal functional strains used for encapsulation in this study (*Lactobacillus crispatus* BC4 and *Lactobacillus gasseri* BC9) belong to the collection of the Department of Pharmacy and Biotechnology (FABIT, University of Bologna, Bologna, Italy) and were isolated from the vaginas of pre-menopausal Caucasian women (aged 18–45 years), with no symptoms of vaginal or urinary tract infections, in accordance with the Ethics Committee of the University of Bologna (52/2014/U/Tess). *Lactobacillus delbrueckii* subsp. *bulgaricus* and *Streptococcus thermophilus* which were used for the fermentation of the soy beverage, were provided by Sacco srl (Cadorago, Italy). Fresh cultures of each strain were obtained from frozen stocks by two consecutive transfers in MRS broth (Oxoid, Basing-stoke, UK) using a 1% (*v*/*v*) inoculum and incubated overnight at 42 °C in aerobic conditions.

### 2.2. Production of Microcapsules

Encapsulated *L. crispatus* BC4 and *L. gasseri* BC9 were prepared according to D’Alessandro et al. [25]. Briefly, the vaginal strains were individually cultured overnight at 37 °C in 1 L MRS broth enriched with 0.05% L-cysteine in an anaerobic jar with AnaeroGen bag (Oxoid). A final concentration of at least 10^9^ cfu/mL was achieved for each strain. Cell counts of the strains were determined after serial dilutions in saline solution (0.9% NaCl) by plating on MRS agar with 0.05% L-cysteine and subsequent incubation at 37 °C for 48 h under anaerobiosis.

One liter of each stock culture was centrifuged at 14,514× *g* for 15 min at 4 °C (Avanti J-26 XP with Ja A-10 rotor, Beckman Coulter, Brea, CA, USA). After removal of the supernatant, the microbial pellet was washed with 1 L of saline solution and then resuspended in 500 mL of a commercial soy beverage with the following composition: 9.04% total solids, 9.8° Brix, pH 6.64, 1.8% lipids, 2.8% carbohydrates, 3% proteins and 0.4% fibers. Spray drying was performed with a mini spray dryer (B191, Buchi-Labortechnik AG, Flawil, Switzerland) equipped with a single liquid nozzle. Temperatures were set at 110 °C for the inlet and 70 °C for the outlet. The pump rate was kept between 19% and 36% aspiration, while the flow rate was 10 mL/min. For each culture, 100 mL of the suspension was spray-dried to obtain an average of 5.2 g powder/100 mL suspension. The spray-dried powder samples were collected from the cyclone, gently mixed, and vacuum-packed in nylon/polyethene 102 μm high-barrier plastic bags (Tecnovac, San Paolo D’Argon, Bergamo, Italy) using an S100 Tecnovac device.

### 2.3. Preparation of Fermented Soy Beverages and Assessment of Viability of Microbial Strains

The production of the fermented soy beverages was carried out in lab conditions as described by [25] using a commercial starter culture (*L. delbrueckii* subsp. *bulgaricus* and *S. thermophilus*) and the vaginal probiotics, encapsulated or not, according to the following plan:

- C: control, fermented soy beverage containing starter cultures only;
- BC4: fermented soy beverage with starter cultures and *L. crispatus* BC4;
- BC9: fermented soy beverage with starter cultures and *L. gasseri* BC9;
- BC4 + BC9: fermented soy beverage containing starter cultures and *L. crispatus* BC4 + *L. gasseri* BC9;
- E-BC4: fermented soy beverage with starter cultures and encapsulated *L. crispatus* BC4;
- E-BC9: fermented soy beverage with starter cultures and encapsulated *L. gasseri* BC9;
- E-BC4 + BC9: fermented soy beverage with starter cultures and encapsulated *L. crispatus* BC4 + *L. gasseri* BC9.

After their preparation, the viability of starter cultures and vaginal strains in these fermented soy beverages was determined after 1 day of storage, using the methods proposed by D’Alessandro et al. [25].

### 2.4. In Vitro Digestion

Fermented soy beverages were subjected to in vitro digestion according to the INFOGEST protocol [26]. In vitro digestion lasted for 242 min consisting in 2 min of oral digestion, 120 min of gastric digestion, and 120 min of intestinal digestion, at 37 °C. During the process, several consecutive enzymatic reactions took place by adding simulated saliva, simulated gastric juice (at pH 3, containing 2000 U/mL pepsin), and simulated pancreatic juice (at pH 7, including 10 mM bile and 100 U/mL pancreatin, Sigma-Aldrich, Schnelldorf, Germany). Samples were taken at the end of the duodenal phase. After digestion, the samples were immediately frozen at −80 °C and then lyophilized.

### 2.5. Volunteers and Fecal Sample Collection

Fecal samples were obtained from 3 post-menopausal women (55 ± 2 years old) and collected at IPLA-CSIC (Oviedo, Spain). All participants followed an unrestricted diet and had not taken antibiotics during the previous 6 months. An informed written consent was obtained from each volunteer, and the study was approved by the Regional Committee of Ethics on Research of the Principality of Asturias (ref. CEImPA 2022.181). Fresh samples were collected, immediately introduced into anaerobic jars (Anaerocult A System, Merck, Darmstadt, Germany), transported to the laboratory within 1 h, and directly used for the fecal batch cultures.

### 2.6. Fecal Batch Cultures

Predigested soy beverage products obtained after INFOGEST digestion were then added into independent bottles inoculated with fecal slurries of post-menopausal women. pH-uncontrolled fecal batch fermentations were performed in basal medium (BM), according to [27] with minor modifications. BM and sterile phosphate-buffered saline (PBS) solutions were prepared and reduced overnight in an anaerobic chamber before fecal sample processing. On the day of the assay, fecal samples were placed in anaerobic conditions, centrifuged, washed, and re-suspended in PBS to a concentration of 1/10 (*v*/*v*). Pre-reduced BM was inoculated with the fecal homogenate (10% *v*/*v*) and distributed into bottles of the ANKOM RF system (Ankom Technology, Macedon, NY, USA) to a final volume of 80 mL per bottle. The fecal cultures were then allowed to stabilize overnight under anaerobic conditions at 37 °C before the addition of a 5% (*v*/*v*) predigested fermented soy beverage solution (0.15 g of lyophilized digested product dissolved in 5 mL of water). Bottles with different digested soy beverage products as well as a negative control (water) were incubated under anaerobiosis at 37 °C for 24 h. Samples (2 mL) were taken in duplicate at time 0 before incubation (basal conditions: baseline) and after 24 h of incubation. The samples were centrifuged at full speed for 15 min, and pellets and supernatants were stored separately at −20 °C until analysis.

### 2.7. pH and Gas Monitoring in Fecal Cultures

The pH of fecal cultures was determined with a pH meter SensION + PH3 (HACH, Barcelona, Spain). The cumulative gas produced during the different fermentation conditions was monitored in real time by using the ANKOM RF system and the mL of gas produced calculated as indicated by [27].

### 2.8. Short-Chain Fatty Acids (SCFAs) Quantification

The analysis of SCFAs (acetic, propionic and butyric acids) was performed by gas chromatography with flame ionization detector (GC-FID) in the fecal culture supernatants (CS). Briefly, 0.25 mL of the culture supernatants were mixed with 0.3 mL methanol, 0.05 mL of an internal standard solution (2-ethylbutyric 1.05 mg/mL), and 0.05 mL of 20% formic acid. This mixture was centrifuged and injected into a GC as described by [27]. Samples were analyzed in triplicate. Absolute levels in molar concentration of SCFAs were calculated for each fermentation batch with the different digested products tested.

### 2.9. Determination of Microbiota Composition

DNA was extracted from the bacterial pellets by using the QIAamp DNA Stool Mini kit (Qiagen GmbH, Hilden, Germany) according to Nogacka et al. [27] and the isolated DNA was stored at −20 °C for subsequent qPCR analyses and 16S rRNA gene sequencing. Absolute levels of some relevant intestinal microbial groups such as *Lactobacilli*, *Bifidobacteria* and *Enterobacteriaceae* were determined at 0 and 24 h of fermentation by qPCR using primers and conditions according to Dao et al. [28]. Purified DNA was also used as a template for amplification of partial 16S rRNA at Novogene Bioinformatics Technology Co., Ltd. (Cambridge, UK). Briefly, DNA concentration was determined in a Qubit 3.0 fluorometer (Life Technologies, Carlsbad, CA, USA) and used for amplification of the hypervariable V3-V4 region with primers 341F (CCTAYGGGRBGCASCAG) and 806R (GGACTACNNGGGTATCTAAT) following the Illumina-recommended “16S Metagenomic Sequencing Library Preparation” protocol. Then, the amplicons were sequenced on an Illumina NovaSeq 6000 platform. And by using samples’ unique barcodes, raw 250 bp paired-end reads were assigned to the different samples and merged to raw tags by using FLASH (Version 1.2.7) [29]. After quality filtering and chimera removal, the tags were compared with the reference SILVA 138 database. ASV denoise with DADA2 as reported by Callahan et al. [30] and subsequent species annotation and phylogenetic relationship construction were performed in the QIIME2 software (Version QIIME2-202006) [31] to obtain initial ASVs (Amplicon Sequence Variants) [32]. The absolute abundance of ASVs was normalized using a standard sequence number corresponding to the sample with the least sequences. Subsequent analyses in QIIME2 on the normalized data were performed.

### 2.10. Statistical Analysis

All experimental data are reported as mean ± standard deviation. The obtained data were analyzed by Statistica software (version 8.0; StatSoft, Tulsa, OK, USA) adopting the analysis of variance (ANOVA), and Tukey’s test for data comparisons. Sequencing OTUs data were presented graphically using the R package pheatmap (v1.0.12).

## 3. Results and Discussion

The study uses a fecal batch culture model to investigate the effects of fermented soy beverages enriched with probiotic vaginal lactobacilli (encapsulated or not) on the fecal microbiota of post-menopausal women. While the short incubation time of the model (24 h) limits the ability to capture long-term microbial interactions, it provides reliable data on the immediate effects of the products as observed in similar studies [33,34,35]. The number of donors (*n* = 3) is another limitation, although it is consistent with sample sizes used in other studies [36,37,38,39,40]. Moreover, pre-clinical evaluation is crucial for testing and screening different ingredients, probiotics and foods before moving on to intervention studies. Despite these limitations, the data obtained could have potential translational value for in vivo conditions by providing preliminary insights into how such interventions may affect human gut microbiota.

### 3.1. Microbial Strain Viability Assessment in Fermented Product

The viability of starter cultures and vaginal strains was evaluated in fermented soy beverage after 1 day of storage. The data obtained for all samples showed cell loads ranging between 6.7 and 7 log cfu/mL for *L. delbrueckii bulgaricus*, 7.5 log cfu/mL for *S. thermophilus*, and between 7 and 7.5 log cfu/mL for *L. crispatus* BC4 and *L. gasseri* BC9 under all conditions tested, in accordance with the results of [25].

### 3.2. pH Variations and Gas Production During Batch Colon Incubation

**Figure 1:**
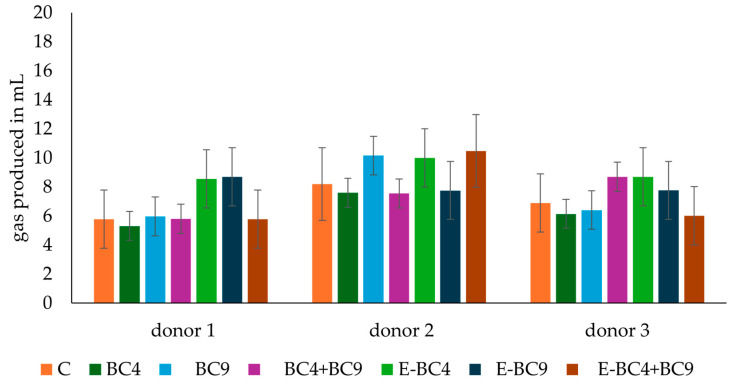
Cumulative gas produced (mL) after 24 h of incubation in fecal cultures with female post-menopausal microbiota of donors 1, 2 and 3. Statistical analysis was performed using a one-way ANOVA with Tukey’s post hoc test comparing different donors within each sample and the same sample among different donors. No significant differences were found in either comparison (*p* > 0.05).

pH variations and gas production of the batch colon incubations are reported in Table 1 and Figure 1, respectively. In accordance with previous studies [27,41], a certain heterogeneity was observed when the fecal cultures from the different donors were incubated with the predigested soy beverage. Overall, a pH decrease was observed in both donor 2 and 3 after 24 h, while an increase was observed in donor 1. In the control cultures, the pH changes ranged from 0.1 to −0.16 units depending on the donor, which is in good agreement with those observed in other studies for the control fecal cultures [27]. This reduction in pH is usually associated with bacterial carbon metabolism which produces short-chain fatty acids and lactic acid [42], while pH increase is associated with nitrogen metabolism (protein fermentation) once sugars are completely fermented by microorganisms. Therefore, the differences observed among donors are likely due to differences in the metabolic properties of their microbiotas, with that of donor 1 being more prone to protein compared to carbohydrate fermentation.

Together with the pH, the gas production was measured for assessing the metabolic activity of the microbiotas in the fecal cultures. The pressure increase (psi) data of the fecal cultures were converted to mL of gas produced (Figure 1). Cumulative gas production in control cultures, ranging from 5.8 to 8.2 mL depending on the donor, underlines the physiological interindividual variability in line with the work of Nogacka et al. [29]. With regards to the fecal cultures incubated with digested soy products with vaginal strains, encapsulated or not, showed no significant differences between donors or samples in terms of gas production (*p* > 0.05). Notably, the drop in pH and the amount of gas produced under the majority of the different experimental conditions tested did not show any large differences among them. These changes are smaller than those reported in previous studies, carried out in the same model, in which additional carbon sources (prebiotics) were added [27,41]. This limited drop in pH and gas produced indicates a limited availability of fermentable substrates in the digested soy beverages tested.

### 3.3. Production of Short-Chain Fatty Acids (SCFAS)

It is well established that the gut microbiome plays a key role in human health through the production of short-chain fatty acids (SCFAs) [43,44,45]. Acetate, propionate, and butyrate, the most important SCFAs, fulfill important physiological functions. Acetate can act directly on the gut microbiota as a carbon source for some bacteria or it can be absorbed by the host and transported to the liver, where it serves to synthesize cholesterol and fatty acids and plays a major role in promoting ileal motility [46]. When absorbed by the host, propionate lowers serum cholesterol levels, lipogenesis and cancer risk [47] and reduces the etiology and progression of metabolic syndromes [48]. Butyrate, the main source of energy for colonocytes, stimulates the differentiation of colonic epithelial cells, promotes mucin production and improves the integrity of tight junctions [46,49,50]. The concentration (expressed in mM) of total SCFAs and individual SCFAs (acetate, propionate, butyrate) and branched chain fatty acids (BCFAs: isovalerate, isobutyrate) during batch colonic incubation are reported in Table 2, for the three different donors.

As indicated in Table 2, acetate was, as expected, the most abundant substance, followed by butyrate and propionate. Acetate, propionate, and butyrate should actually occur in the colon in a molar ratio of 3:1:1 [46]. In donor 1, the highest acetate and butyrate content (25.74 and 6.11 mM, respectively) was reached after 24 h in samples incubated with E-BC4 + BC9, while the propionate content was higher in E-BC9 (5.09 mM) (*p* < 0.05). In donor 2, all three major SCFAs were higher in BC4 (20.21, 4.34, and 6.03 mM), followed by E-BC4 + BC9 (19.25, 4.08, and 5.73 mM, for acetate, propionate, and butyrate, respectively) after 24 h simulated colonic incubation (*p* < 0.05). In donor 3, the highest values for acetate were observed in the sample with E-BC9 (21.89 mM) followed by E-BC4 + BC9 and BC4 (20.81 and 20.46 mM, respectively) (*p* < 0.05). Propionate was higher in E-BC9 (7.08 mM), while butyrate reached the highest values in the samples with E-BC9 (6.67 mM) and BC4 (6.35 mM). In this case, however, the control (C) also showed a high butyrate content (5.94 mM) after 24 h of incubation, which was not significantly different from that of E-BC9 and BC4 (*p* > 0.05). Since the initial concentrations of the SCFAs were different among samples, the difference in their production was also calculated. In donor 1, E-BC4 + BC9 was still the sample with a higher increase in acetate and butyrate compared to control. In donor 2, acetate increased in BC4 and E-BC4 + BC9, while propionate and butyrate mainly increased in BC4, E-BC4 and E-BC4 + BC9. In donor 3, acetate and propionate increased mainly in BC4 and E-BC4 + BC9, while butyrate increased especially in BC4. Comparison of SCFAs levels among donors at time zero (Table 2) allowed us to observe inter-individual differences. For instance, regardless of the fecal culture tested, donor 1 showed the highest levels of acetate whereas donor 3 exhibited the same for propionate, with butyrate showing variability depending on the probiotics added and the volunteer. Moreover, higher basal levels of certain SCFAs, such as acetate for donor 1 or propionate for donor 2, seem to be associated with a higher production of these same metabolites during incubation. These results suggest the existence of different metabolic types among the donors’ microbiotas. In addition to the SCFAs mentioned above, the intestinal microbiota produces considerably lower amounts of isobutyric acid, isovaleric acid, commonly known as branched short-chain fatty acids (BCFAs) derived from branched chain amino acids (valine, leucine, and isoleucine) generated from undigestible protein reaching colon. A diet high in protein and low in complex carbohydrates (typical of the Western diet), increases levels of BCFAs in vitro [51], both in animal and human intervention studies [52,53]. Conversely, supplementation with complex carbohydrates, capable of reaching the colon, tends to lower fecal BCFAs levels [3]. In our experimental work, the BCFAs content differed significantly from the control samples. Interestingly, the control culture from donor 1 showed slightly higher levels of the analyzed BCFAs than those from the other donors. This may be indicative of a larger degree of aminoacids fermentation in this volunteer, which may be linked to the previously discussed changes in the pH of the culture during incubation. Taking into account the physiological variability observed among the different donors, as also shown by looking at the calculated theoretical ratios between acetate, propionate and butyrate (Table 2), the overall results in terms of SCFAs showed that *L. crispatus* BC4 or its encapsulated forms (BC4, E-BC4) together with *L. gasseri* BC9, especially when encapsulated alone or with BC4 (E-BC4, E-BC4 + BC9), had a greater effect on metabolic activity than the control or other samples. As emphasized in the discussion of the results, encapsulation of vaginal strains has been shown to increase metabolic activity under certain experimental conditions compared to their non-encapsulated counterparts. This improvement can be attributed to the encapsulation process, which could not only improve microbial survival rates, but also enable a controlled release of microorganisms. Such controlled release is crucial as it allows for more effective interaction with the gut microbiota, thus increasing metabolic activity over time [54,55]. In addition, the encapsulation matrix itself provides a protective microenvironment that further supports higher metabolic performance during colonic fermentation. It is important to also consider the strain-specific responses to the encapsulation process, as different strains may interact with the matrix in different ways, leading to different metabolic outcomes for different probiotic strains, as already shown in several studies [56,57].

### 3.4. Microbiota Composition

#### 3.4.1. Quantitative PCR Analyses

Among the commensal bacteria that reside in the human gastrointestinal tract (GIT), bifidobacteria are key “probiotic” microbes in the human GIT [58,59,60,61,62], though they make up less than 10% of the microbiota, with their levels influenced by factors such as age, diet, and gender (50–51). Similarly, *Lactobacillus* species, though less abundant, are crucial for gut health and are often associated with beneficial outcomes [63]. While probiotic interventions have shown positive effects, the specific role of lactobacilli remains under investigation. Furthermore, the remarkable variation in intestinal abundance of this genus between healthy and diseased, or health-compromised, individuals suggests that *Lactobacillus*, or at least certain species or genotypes of *Lactobacillus*, may be useful gut biomarkers [64]. In contrast, an increase in *Enterobacteriaceae* has also been associated with chronic ulcerative colitis (UC) inflammation compared to acute status, showing a positive correlation of this bacterial family with severe disease stage [65]. These studies support the notion that *Enterobacteriaceae* seem to have a growth advantage over other members of the gut microbiota commensals in the inflamed GIT mucosa.

These considerations prompted us to determine the levels of these microbial groups by qPCR to understand the response of these intestinal populations, which are part of the microbiota of post-menopausal women, to supplementation with our fermented soy beverages. The absolute concentrations (Log DNA cells/g fecal culture) of *Bifidobacterium* spp., *Lactobacillus* spp., and the *Enterobacteriaceae family* were determined at 0, 24 of the incubation of the fecal cultures from donor 1, donor 2 and donor 3 (Table 3).

An analysis of the bifidobacteria data showed that there was an interindividual variability between the different donors. In donor 1, the addition of the digested soy beverages containing vaginal probiotics showed no significant effect on the concentration of bifidobacteria, both in terms of initial time and final time. The deltas also showed no significant differences between the different experimental conditions. In donor 2, which started with a lower concentration of bifidobacteria with respect to donors 1 and 3, a general increase in the concentration of these microorganisms was observed. In particular, the addition of the sample containing BC4 + BC9 resulted in the highest load (8.44 log cells/g). However, a look at the deltas shows that the sample with E-BC9 was also able to significantly increase the level of bifidobacteria (Δlog 3.69). Donor 3 was the sample with the highest bifidobacteria load already at the beginning of the incubation. For this reason, no increase in bifidobacteria was observed, but on the contrary, there was a slight decrease, especially in the samples with BC4 + BC9 and E-BC4 + BC9. The *Lactobacillus* cell load was then analyzed. Two out of three donors showed concentrations of about 4.2 in the control, while donor 2 showed values below the limit of quantification (3.5 log cells/g). The addition of the samples containing probiotics led to an increase in the level of lactobacilli in donors 1 and 3 regardless of the experimental condition tested. However, a decrease over time (24 h) was then observed in all samples except the control (donors 1 and 3) and BC4 in donor 1. In donor 2, only two samples supplemented with vaginal probiotics showed an increase in lactobacilli after 24 h, such as BC4 + BC9 and E-BC9. Finally, the concentration of *Enterobacteriaceae* was evaluated. Table 3 shows that the concentrations in donors 1 and 3 were again more similar at approx. 8 log cells/g in the control. While no significant changes in the concentration of *Enterobacteriaceae* were observed in donor 1 after 24 h of incubation, a general decrease was observed in donor 3, especially in BC9, BC4 + BC9, E-BC4, and E-BC4-BC9. Donor 2 was the one with the lowest level of *Enterobacteriaceae* and a general increase was observed there in all samples except the control.

In general, it seems evident that the addition of probiotics under certain experimental conditions initially increased the total number of *Lactobacillus* bacteria, but this led to a greater decrease after 24 h (e.g., donor 1 with E-BC4 ∆ 24-0, −1.97 for *Lactobacillus*). This highlights the complex and dynamic balance between different microbial groups within the intestinal ecosystem. The gut microbiota is influenced by numerous factors, including diet, genetics, environmental influences, and interactions between microbial species [66]. This delicate balance can shift in response to external interventions, such as the introduction of probiotics. While probiotics may initially promote the growth of beneficial microbes such as *Lactobacillus*, their long-term effects may vary and lead to fluctuations in microbial populations over time. Other authors [67] have shown that probiotics can initially significantly increase the growth of lactic acid bacteria, including *Lactobacillus,* in the fecal bacterial flora of piglets, but then decrease it after 24 h, suggesting a transient effect of probiotics on microbial balance. This emphasizes the importance of considering not only the immediate effects of probiotic supplementation, but also the necessity for continuous intake to achieve a stable effect on the gut microbiota [68]. Given the complexity of microbial interactions, regular intake of probiotics may be crucial for maintaining a favorable balance and avoid short-term imbalances that could undermine the desired therapeutic effects.

#### 3.4.2. Effect of Digested Soy Beverages Containing Encapsulated and Non-Encapsulated Probiotics on the Microbial Community Composition in Short-Term Colonic Incubations

**Figure 2:**
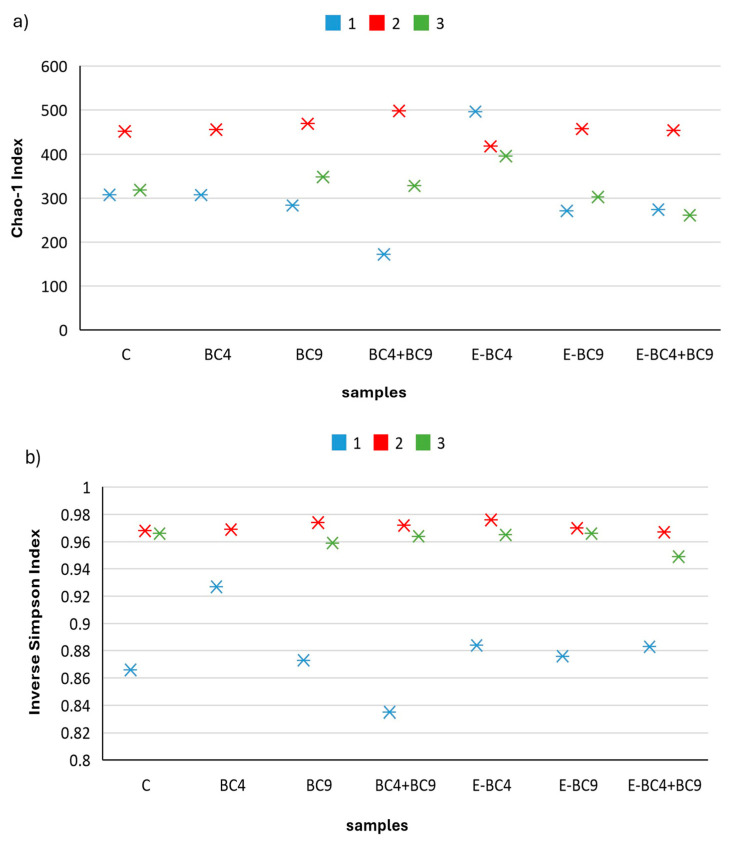

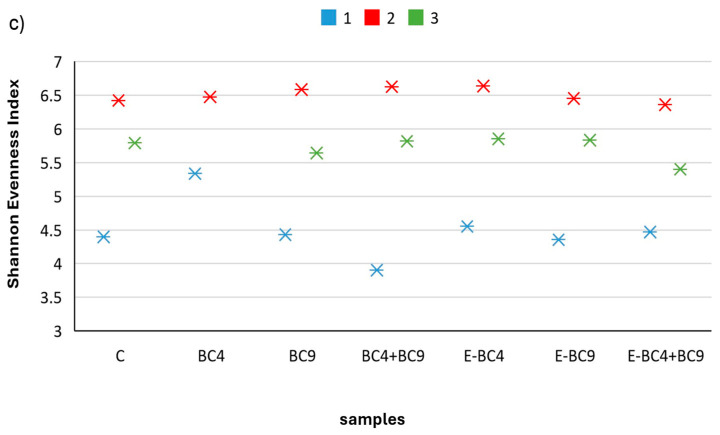
Microbiota diversity indices such as richness (Chao-1) (**a**), diversity (Inverse Simpson) (**b**), and evenness (Shannon) (**c**), observed for the predigested soy beverages with different vaginal probiotics, encapsulated or not, on the microbial population of post-menopausal women (1: donor 1, 2: donor 2, and 3: donor 3).

The microbiota diversity indices ((richness (Chao-1), diversity (Inverse Simpson), and evenness (Shannon)) were analyzed to study the impact of the predigested soy beverages containing different vaginal probiotics, encapsulated or not, on microbial population of pre-menopausal women (Figure 2).

Overall, richness (Chao-1), diversity (Inverse Simpson), and evenness (Shannon) of donor 2 were the highest among donors, followed by donors 3 and 1, that showed the lowest scores. This is in line with the fact that there is an intrinsic variability among donors and for this reason they were described separately. In donor 1, richness was maintained comparable to the control in most samples while it decreased and increased in sample BC4 + BC9 and E-BC4, respectively. Overall, the evenness (Shannon) and diversity (Simpson) of the samples were similar to the control except for BC4 and BC4 + BC9. In donor 2, a slight increase in the evenness in BC9, BC4 + BC9 and E-BC4 was observed compared to the untreated one. With regard to richness, all the samples presented values comparable to control, except for sample BC4 + BC9 which presented the highest value and E-BC4 where the value decreased. In donor 3, the richness decreased in E-BC4 + BC9 and increased in E-BC4 with respect to the control. Regarding evenness and diversity, these values were reduced in E-BC4 + BC9 compared to control. All these results suggest that samples, especially containing E-BC4 + BC9, E-BC4, and BC4 + BC9, had an impact on the microbial composition of the fecal sample with respect to the other experimental conditions tested. Looking at single product tested, the mix of encapsulated probiotics was the one with a stronger impact on the microbiota because it reduced evenness and the diversity of donor 3. Donor 1 was mainly impacted by the non-encapsulated mix that reduced all three parameters.

**Figure 3:**
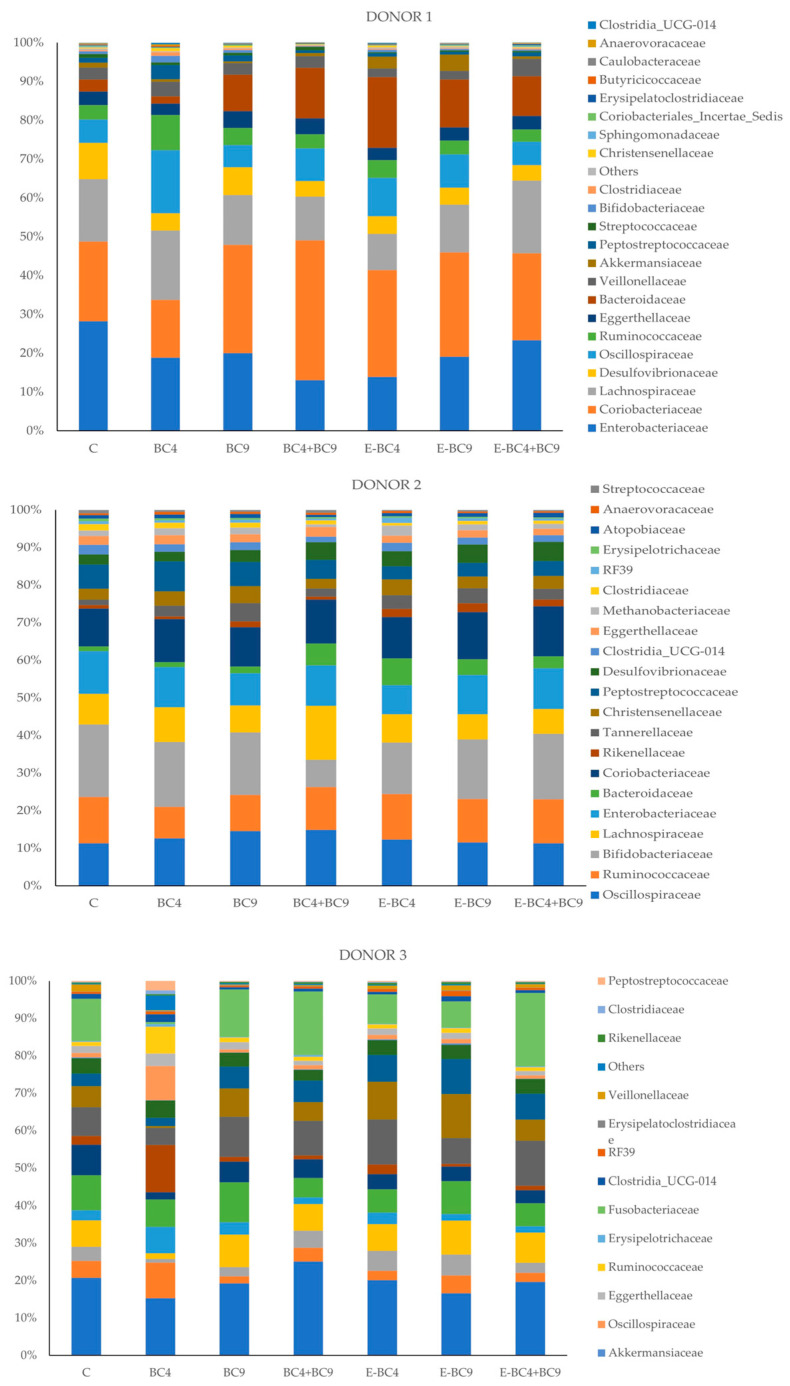
Relative abundance of bacteria at the family level at 24 h of incubation in fecal cultures of donor 1, 2 and 3 with the different predigested soy beverages containing or not encapsulated (E) or non-encapsulated vaginal probiotics (C: Control; 4: *L. crispatus* BC4; 9; *L. gasseri* BC9; 4 + 9: mix of BC4 and B9).

In more detail, as shown in Figure 3, at the family level in donor 1 we observed lower abundance of *Enterobacteriaceae* and *Desulfovibrionaceae* in the experimental conditions with vaginal probiotics compared to control. Some of the main changes were observed using the sample with non-encapsulated probiotic BC4 since it showed a higher abundance of *Ruminococcaceae*, *Peptostreptococcaceae*, *Bifidobacteriaceae*, *Clostridiaceae* and *Oscillospiraceae*. When strain BC4 was encapsulated, a higher abundance of *Bacteroidaceae* and *Akkermansiaceae* was observed. On the contrary, especially the mix of BC4 + BC9 increased the relative abundance of *Coriobacteriaceae*. The relative abundance of *Akkermansiaceae* was further increased when probiotic BC4 and BC9 were used encapsulated. In donor 2, *Ruminococcaceae* were reduced in samples with strain BC4 and BC9 non-encapsulated, *Lachnospiraceae* were higher when using BC4 + BC9, *Bacteroidaceae* were higher in samples containing encapsulated bacteria or non-encapsulated mix, *Tannerellaceae* were all higher than control regardless of the bacteria applied, *Peptostreptococcaceae* abundance did not increase when using the probiotics encapsulated, and *Desulfovibrionaceae* mainly increased when using the mix. In donor 3, looking at the single treatments and comparing them to the control, it is possibile to observe a lower relative abundance of *Lachnospiraceae*, *Bifidobacteriaceae*, *Tannerellaceae* and *Acidaminococcaceae* when using strain BC4, while it increased the relative abundance of *Enterobacteriaceae*, *Oscillospiraceae*, *Ruminococcaceae*. On the contrary, *Desulfovibrionaceae*, *Marinifilaceae* and *Bacteroidaceae* increased when using encapsulated bacteria.

**Figure 4:**
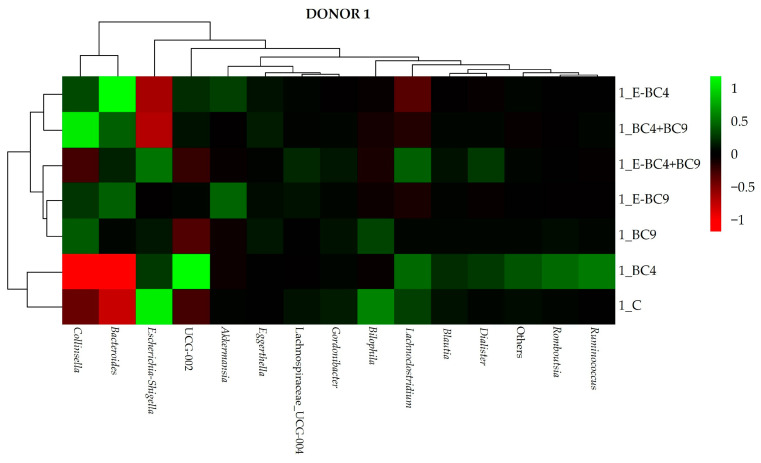
Heatmap showing the abundance of bacteria at genus level at 24 h of incubation in fecal cultures of donor 1 with the different predigested soy beverages containing or not encapsulated (E) or non-encapsulated vaginal probiotics.

Heatmap of the bacterial genera in the samples obtained after the different treatments for the cultures of donor 1 (Figure 4) showed that sample BC4 clustered together with the control. Samples with strain BC9, encapsulated or not, clustered together and were characterized by a higher abundance of *Collinsella*. However, the highest abundance of *Collinsella* was observed in sample E-BC4 and BC4 + BC9 that also presented a high abundance of *Bacteroides*. Eventually, sample E-BC4 and E-BC9 had the highest abundance of *Akkermansia*.

**Figure 5:**
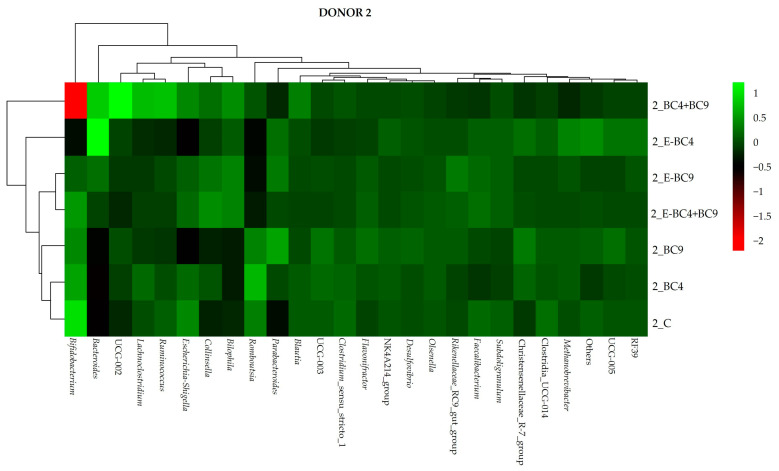
Heatmap showing the abundance of bacteria at genus level at 24 h of incubation in fecal cultures of donor 2 with the different predigested soy beverages containing or not encapsulated (E) or non-encapsulated vaginal probiotics.

Looking at donor 2 (Figure 5), sample with BC4 + BC9 was the most differentiated one with the lowest abundance of *Bifidobacterium.* On the other hand, the use of encapsulated BC4 + BC9 determined the highest abundance of *Collinsella* and *Bilophila*. With the addition of E-BC4 and BC4 + BC9 an increase in *Bacteroides* was observed.

**Figure 6:**
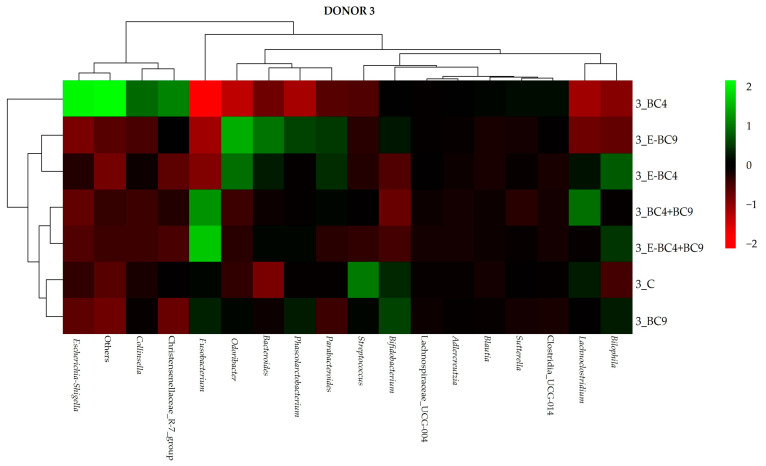
Heatmap showing the abundance of bacteria at genus level at 24 h of incubation in fecal cultures of donor 3 with the different predigested soy beverages containing or not encapsulated (E) or non-encapsulated vaginal probiotics.

Regarding donor 3 (Figure 6), the presence of probiotic bacteria in the formulation confirmed the increase in the relative abundance of *Fusobacterium* (except for BC4, E-BC9, E-BC4), *Bilophila*, *Odoribacter* (in encapsulated probiotics such as E-BC4, E-BC9 and E-BC4, E-BC4 + BC9, respectively) and *Bifidobacterium* (only in samples BC9 and E-BC9) and the reduction in *Christensenellaceae R-7group* (except for BC4). Only the addition of probiotics (with the exception of BC4) led to an increase in the relative abundance of the genus *Bacteroides* compared to the control. Regarding the scientific literature on the gut microbiota of post-menopausal women, it is important to emphasize the heterogeneity of results between studies, which could be related to different sample sizes and different study populations, as the gut microbiome is known to vary according to geography and lifestyle [3]. Although various gut bacterial taxa were reported to differ by menopause status, some consistent results were observed across studies. For example, several studies found a lower abundance of *Firmicutes* and *Ruminococcus* [69,70,71] and a higher abundance of *Butyricimonas* [72,73], *Dorea* [71,73], *Prevotella* [3,71], *Sutterella* [3,73], and *Bacteroides* [3,71] genera or species in post-menopausal women compared to pre-menopausal women.

As the functions and health effects of these bacteria are not fully understood, it is difficult to completely assess whether menopause could have a negative impact on the composition of the gut microbiome based on the available information. For example, it has been reported that ruminococci, which can play an important role in the production of short-chain fatty acids in the gut [74], are less common in the post-menopausal phase. Interestingly, in our study, the sample with the probiotic BC4 showed a higher abundance of *Ruminococcaceae* in donors 1 and 3. Other observed menopause-related groups that could have health effects are *Akkermansia*, particularly *Akkermansia municiphilia* which is known for its beneficial effects on metabolic and immune system in humans [75,76,77], is generally reduced in the post-menopausal stage [3]. In this context, samples E-BC4 and E-BC9 in donor 1 showed the highest abundance of *Akkermansia*. In addition, according to [78], *Odoribacter* genus was more abundant in post-menopausal women than in pre-menopausal women. Increased odoribacteria in post-menopausal women lead to increased levels of short-chain fatty acids (SCFAs), hydrogen, and hydrogen sulfide [79,80]. SCFAs increase fatty acid oxidation and energy metabolism, are involved in the synthesis of serotonin and the stabilization of neurons, and increase circulating insulin-like growth factor-1, which stimulates osteogenesis [81]. Thus, an odoribacter-induced increase in SCFAs may reduce the risk of obesity, hyperlipidaemia, depression and osteoporosis in post-menopausal women. In contrast, increased hydrogen sulfide production leads to inflammatory reactions [78]. *Odoribacter,* therefore, has both positive and negative effects (similar to the effects of post-menopausal syndrome) [82]. In our study, the presence of E-BC4 and E-BC9 confirmed the increase in the relative abundance of *Odoribacter* in donor 3. It is also interesting to note that in all three donors, especially in the presence of the probiotics, (especially E-BC4, E-BC9, BC4 + BC9) there was an increase in *Bacteroides*. *Bacteroides* are common in menopausal women and their role in the gut can also be either beneficial or harmful, depending on other microbiome and host factors [3,83].

## 4. Conclusions

This study highlights the effects of fermented soy beverages enriched with probiotic vaginal lactobacilli on the fecal microbiota of post-menopausal women using a simple fecal batch culture model. In addition, the experimental work had the ambition to evaluate the effects of process variables such as microbial encapsulation to find strategies for increasing the delivery of vaginal lactobacilli into the gut and consequently into the vaginal ecosystem. Although the mechanisms are not always clear, the presence of vaginal lactobacilli can, in certain conditions, increase the level of beneficial microbial population in fecal samples. An inter-individual variability related to the response of the microbiota to the product tested was observed. For instance, in donor 2, which started with a lower concentration of bifidobacteria compared to donors 1 and 3, a general increase in the concentration of these microorganisms was observed, especially in the presence of BC4 + BC9 and E-BC9. The sample with probiotic BC4 showed a higher abundance of *Ruminococcaceae* in donors 1 and 3, while *Akkermansia* showed a higher abundance in samples E-BC4 and E-BC9 with donor 1. Despite this donor inter-individual variability, the overall results in terms of SCFAs production showed that *L. crispatus* BC4 or its encapsulated form (BC4, E-BC4) together with the encapsulated *L. gasseri* BC9 (E-BC4 + BC9), had a greater impact on metabolic activity than the control (C) or the other samples. In contrast, the decrease in pH and the amount of gas produced under most of the different experimental conditions (with or without the different probiotics) showed no significant differences.

In conclusion, it is important to note that our data could be further strengthened by considering alternative dosages (increased intake of the functional product during its shelf life) or by increasing the cell load of vaginal strains in the original soy beverage. Indeed, regular probiotic intake is crucial for ensuring their effectiveness. In support of this, we have proposed the development of a food product containing functional vaginal strains to improve women’s health. This approach could represent an innovative nutritional strategy that enhances consumer acceptance and promotes consistent, enjoyable probiotic consumption. However, due to the limitations of using a batch culture model and the number of donors recruited in this study (*n* = 3), we cannot generalize our observations. Further studies, including human intervention studies, would be required to substantiate the beneficial effects of these fermented products.

## Figures and Tables

**Table 1:** Decreases in pH (∆ pH) after 24 h of batch incubation using fecal slurries of three female post-menopausal women (donors 1, 2 and 3). Differences are shown for each donor after 24 h of incubation. Statistical analysis was performed by a one-way ANOVA with Tukey’s post hoc test comparing the different samples within each donor and the same sample between different donors (*p* < 0.05). Different lower case letters indicate significant differences between samples within the same donor. Different capital letters indicate significant differences between the same sample among different donors.

Samples	∆ pH Donor 1	∆ pH Donor 2	∆ pH Donor 3
C	0.10 (±0.02) ^aA^	−0.16 (±0.01) ^aB^	−0.05 (±0.03) ^aC^
BC4	0.11 (±0.02) ^aA^	−0.12 (±0.02) ^aB^	−0.11 (±0.02) ^abB^
BC9	0.10 (±0.01) ^aA^	−0.11 (±0.03) ^aB^	−0.14 (±0.01) ^bB^
BC4 + BC9	0.13 (±0.02) ^aA^	−0.12 (±0.02) ^aB^	−0.06 (±0.03) ^aC^
E-BC4	0.09 (±0.02) ^aA^	−0.18 (±0.04) ^aB^	−0.11 (±0.03) ^abB^
E-BC9	0.11 (±0.02) ^aA^	−0.16 (±0.02) ^aB^	−0.10 (±0.03) ^abB^
E-BC4 + BC9	0.08 (±0.03) ^aA^	−0.29 (±0.02) ^bC^	−0.12 (±0.03) ^abB^

**Table 2:** Absolute levels (mM) of SCFAs and BCFAs (acetate, propionate, butyrate, isovalerate, isobutyrate, total SCFAs, theoretical ratio calculated between acetate, propionate, butyrate) after 0 and 24 h (and the respective differences Δ24-0) of incubation with the different digested soy beverage products in presence of fecal cultures of donor 1, 2 and 3. Statistical analysis was performed using a one-way ANOVA with Tukey’s post hoc test, comparing within each fermentation time point the different samples within each donor and the same sample between different donors (*p* < 0.05). Different lowercase letters for the same time point indicate significant differences between samples within the same donor. Different capital letters for the same time point indicate significant differences between the same sample between different donors.

	Donor 1			Donor 2			Donor 3		
	0 h	24 h	Δ 24-0	0 h	24 h	Δ 24-0	0 h	24 h	Δ24-0
***Acetate***									
C	21.77 (±0.02) ^fA^	22.37 (±0.04) ^cA^	0.60	10.52 (±0.04) ^bB^	11.68 (±0.02) ^aB^	1.16	13.89 (±0.07) ^aC^	19.83 (±0.02) ^bC^	5.95
BC4	17.73 (±0.03) ^aA^	18.72 (±0.11) ^bA^	0.99	8.65 (±0.06) ^aB^	20.21 (±0.17) ^fB^	11.56	13.25 (±0.08) ^aC^	20.46 (±0.20) ^cB^	7.21
BC9	18.88 (±0.01) ^cA^	22.43 (±0.19) ^cA^	3.55	11.20 (±0.01) ^cB^	11.48 (±0.04) ^aB^	0.28	13.61 (±0.05) ^aC^	19.84 (±0.04) ^bC^	6.23
BC4 + BC9	18.46 (±0.06) ^bA^	14.20 (±0.08) ^aA^	−4.26	15.26 (±0.04) ^eB^	15.86 (±0.16) ^bB^	0.61	14.51 (±0.01) ^bC^	17.81 (±0.01) ^aC^	3.30
E-BC4	19.68 (±0.03) ^eA^	23.65 (±0.06) ^dA^	3.97	10.98 (±0.07) ^bB^	17.54 (±0.04) ^dB^	6.56	15.87 (±0.16) ^cC^	17.18 (±0.10) ^aC^	1.30
E-BC9	19.26 (±0.01) ^dA^	22.77 (±0.07) ^cA^	3.51	17.56 (±0.01) ^fB^	16.24 (±0.02) ^cB^	−1.32	16.24 (±0.07) ^dC^	21.89 (±0.12) ^dC^	5.65
E-BC4 + BC9	19.22 (±0.05) ^dA^	25.74 (±0.03) ^eA^	6.51	12.77 (±0.08) ^dB^	19.25 (±0.18) ^eB^	6.48	13.39 (±0.01) ^aC^	20.81 (±0.25) ^cC^	7.43
***Propionate***									
C	3.75 (±0.01) ^cA^	4.11 (±0.11) ^bA^	0.36	2.18 (±0.01) ^aB^	2.43 (±0.04) ^aB^	0.25	3.95 (±0.03) ^bC^	5.93 (±0.22^) bC^	1.98
BC4	2.97 (±0.01) ^aA^	3.43 (±0.05) ^aA^	0.47	1.94 (±0.03) ^aB^	4.34 (±0.04) ^dB^	2.41	3.76 (±0.03) ^aC^	6.20 (±0.07) ^bC^	2.44
BC9	3.25 (±0.01) ^bA^	4.12 (±0.05) ^bA^	0.88	2.45 (±0.21) ^bB^	2.44 (±0.03) ^aB^	−0.01	3.94 (±0.01) ^bC^	6.11 (±0.01) ^bC^	2.17
BC4 + BC9	3.17 (±0.01) ^bA^	4.41 (±0.03) ^cA^	1.24	3.35 (±0.11) ^dA^	3.41 (±0.44) ^bB^	0.05	4.08 (±0.03) ^cB^	5.06 (±0.13^) aC^	0.97
E-BC4	3.33 (±0.03) ^bA^	4.31 (±0.01) ^cA^	0.97	2.35 (±0.10) ^bB^	3.81 (±0.21) ^bB^	1.46	4.46 (±0.31) ^dC^	5.17 (±0.01) ^aC^	0.71
E-BC9	3.32 (±0.01) ^bA^	5.09 (±0.03) ^dA^	1.77	3.91 (±0.01) ^eB^	3.67 (±0.18) ^bB^	−0.25	4.72 (±0.22) ^dC^	7.08 (±0.08) ^cC^	2.36
E-BC4 + BC9	3.29 (±0.01) ^bA^	3.90 (±0.11) ^bA^	0.60	2.81 (±0.03) ^cB^	4.08 (±0.06) ^cA^	1.27	3.91 (±0.02) ^bC^	6.47 (±0.30) ^bB^	2.56
***Butyrate***									
C	4.58 (±0.21) ^bA^	4.98 (±0.01) ^cA^	0.40	2.89 (±0.13) ^aB^	3.24 (±0.11) ^aB^	0.35	4.40 (±0.12) ^bA^	5.94 (±0.21) ^bC^	1.54
BC4	3.80 (±0.09) ^aA^	4.06 (±0.03) ^bA^	0.26	2.62 (±0.02) ^aB^	6.03 (±0.07) ^eB^	3.41	4.29 (±0.13) ^bC^	6.35 (±0.05) ^cC^	2.06
BC9	4.26 (±0.22) ^bA^	5.26 (±0.03) ^dA^	1.00	3.42 (±0.01) ^bB^	3.36 (±0.02) ^aB^	−0.06	3.85 (±0.02) ^aC^	5.33 (±0.42) ^bA^	1.49
BC4 + BC9	4.23 (±0.22) ^bA^	3.35 (±0.02) ^aA^	−0.88	4.63 (±0.23) ^cA^	4.54 (±0.03) ^bB^	−0.09	4.14 (±0.22) ^bA^	4.74 (±0.11) ^aB^	0.60
E-BC4	4.31 (±0.31) ^bA^	5.28 (±0.04) ^dA^	0.97	3.25 (±0.14) ^bB^	5.10 (±0.03) ^cB^	1.85	4.71 (±0.03) ^cC^	4.97 (±0.09) ^aB^	0.26
E-BC9	4.40 (±0.11) ^bA^	5.30 (±0.04) ^dA^	0.90	5.72 (±0.01) ^dB^	5.05 (±0.01) ^cB^	−0.67	5.22 (±0.01) ^dC^	6.67 (±0.04) ^cC^	1.45
E-BC4 + BC9	4.33 (±0.04) ^bA^	6.11 (±0.01) ^eA^	1.78	4.00 (±0.21) ^cB^	5.73 (±0.04) ^dB^	1.72	3.99 (±0.21) ^abB^	5.73 (±0.25) ^bB^	1.74
***Isovalerate***									
C	0.76 (±0.01) ^aA^	0.71 (±0.02) ^bA^	−0.05	0.53 (±0.04) ^bB^	0.47 (±0.01) ^aB^	−0.06	0.16 (±0.02) ^aC^	0.29 (±0.01) ^bC^	0.13
BC4	0.66 (±0.01) ^aA^	0.64 (±0.06) ^bA^	−0.02	0.39 (±0.01) ^aB^	0.77 (±0.01) ^bA^	0.38	0.17 (±0.03) ^aC^	0.30 (±0.03) ^bB^	0.13
BC9	0.70 (±0.03) ^aA^	0.74 (±0.02) ^bA^	0.05	0.60 (±0.01) ^bB^	0.51 (±0.01) ^aB^	−0.10	0.14 (±0.01) ^aC^	0.28 (±0.01) ^bC^	0.14
BC4 + BC9	0.66 (±0.01) ^aA^	0.48 (±0.02) ^aA^	−0.18	0.85 (±0.01) ^cB^	0.74 (±0.02) ^bB^	−0.10	0.14 (±0.01) ^aC^	0.22 (±0.01) ^aC^	0.08
E-BC4	0.80 (±0.02) ^abA^	0.88 (±0.01) ^cA^	0.08	0.57 (±0.01) ^bB^	0.83 (±0.01) ^cA^	0.26	0.19 (±0.02) ^abC^	0.24 (±0.01) ^aB^	0.05
E-BC9	0.85 (±0.01) ^bA^	0.93 (±0.01) ^cA^	0.08	1.03 (±0.02) ^dB^	0.80 (±0.01) ^bcB^	−0.23	0.23 (±0.02) ^bC^	0.42 (±0.01) ^cC^	0.19
E-BC4 + BC9	0.67 (±0.01) ^aA^	0.81 (±0.08) ^bcA^	0.14	0.73 (±0.09) ^bcA^	0.90 (±0.02) ^dB^	0.16	0.16 (±0.01) ^aB^	0.30 (±0.01) ^bC^	0.14
***Isobutyrate***									
C	0.29 (±0.02) ^abA^	0.27 (±0.01) ^bA^	−0.02	0.19 (±0.03) ^bA^	0.16 (±0.01) ^aB^	−0.02	0.04 (±0.01) ^aB^	0.10 (±0.01) ^aC^	0.06
BC4	0.26 (±0.01) ^aA^	0.24 (±0.03) ^bA^	−0.02	0.13 (±0.01) ^aB^	0.29 (±0.01) ^cA^	0.16	0.05 (±0.01) ^aC^	0.12 (±0.02) ^aB^	0.07
BC9	0.26 (±0.01) ^aA^	0.29 (±0.01) ^bA^	0.03	0.23 (±0.01) ^bB^	0.20 (±0.01) ^bB^	−0.04	0.04 (±0.01) ^aC^	0.10 (±0.01) ^aC^	0.06
BC4 + BC9	0.27 (±0.01) ^aA^	0.17 (±0.01) ^aA^	−0.10	0.33 (±0.01) ^cA^	0.30 (±0.01) ^cB^	−0.03	0.04 (±0.01) ^aB^	0.07 (±0.03) ^aC^	0.03
E-BC4	0.31 (±0.01) ^bA^	0.35 (±0.01) ^cA^	0.03	0.22 (±0.01) ^bB^	0.33 (±0.02) ^cA^	0.11	0.05 (±0.01) ^aC^	0.09 (±0.01) ^aC^	0.03
E-BC9	0.34 (±0.01) ^cA^	0.36 (±0.01) ^cA^	0.02	0.42 (±0.01) ^dB^	0.33 (±0.03) ^cA^	−0.09	0.09 (±0.01) ^bC^	0.16 (±0.01) ^bB^	0.08
E-BC4 + BC9	0.26 (±0.01) ^aA^	0.32 (±0.03) ^bcA^	0.05	0.29 (±0.02) ^cA^	0.37 (±0.01) ^dA^	0.08	0.05 (±0.01) ^aB^	0.10 (±0.01) ^aB^	0.06
***Total Main SCFAs***									
C	30.10	31.45	1.36	15.59	17.34	1.76	22.24	31.71	9.47
BC4	24.50	26.21	1.72	13.21	30.58	17.38	21.30	33.01	11.71
BC9	26.39	31.82	5.43	17.07	17.28	0.21	21.40	31.28	9.89
BC4 + BC9	25.87	21.97	−3.90	23.24	23.81	0.57	22.73	27.61	4.88
E-BC4	27.33	33.24	5.91	16.57	26.45	9.87	25.05	27.32	2.27
E-BC9	26.98	33.15	6.17	27.19	24.96	−2.23	26.18	35.63	9.45
E-BC4 + BC9	26.84	35.75	8.90	19.58	29.05	9.47	21.28	33.01	11.73
***Theoretical Ratio (Acetate: Propionate: Butyrate)***									
C	5.81; 1.00; 1.22	5.44; 1.00; 1.21		4.83; 1.00; 1.33	4.81; 1.00; 1.33		3.52; 1.00; 1.11	3.34; 1.00; 1.00	
BC4	5.97; 1.00; 1.28	5.46; 1.00; 1.18		4.46; 1.00; 1.35	4.66; 1.00; 1.39		3.52; 1.00; 1.14	3.30; 1.00; 1.02	
BC9	5.81; 1.00; 1.31	5.44; 1.00; 1.28		4.57; 1.00; 1.33	4.70; 1.00; 1.38		3.45; 1.00; 0.98	3.25; 1.00; 0.87	
E-BC4	5.82; 1.00; 1.33	3.22; 1.00; 0.76		4.56; 1.00; 1.33	4.65; 1.00; 1.33		3.56; 1.00; 1.01	3.52; 1.00; 0.94	
BC4 + BC9	5.91; 1.00; 1.29	5.49; 1.00; 1.23		4.67; 1.00; 1.38	4.60; 1.00; 1.34		3.56; 1.00; 1.06	3.32; 1.00; 0.96	
E-BC9	5.80; 1.00; 1.33	4.47; 1.00; 1.04		4.49; 1.00; 1.46	4.43; 1.00; 1.38		3.44; 1.00; 1.11	3.09; 1.00; 0.94	
E-BC4 + BC9	5.84; 1.00; 1.32	6.60; 1.00; 1.57		4.54; 1.00; 1.42	4.72; 1.00; 1.40		3.42; 1.00; 1.02	3.22; 1.00; 0.89	

**Table 3:** Absolute levels (Log DNA cells/g fecal culture) of *Bifidobacterium* genus, *Lactobacillus* genus and *Enterobacteriaceae* family determined by qPCR after 0, 24 h of incubation with the different digested soy beverage products in presence of fecal cultures of donor 1, 2 and 3.

		Donor 1			Donor 2			Donor 3		
		0 h	24 h	Δ24-0	0 h	24 h	Δ24-0	0 h	24 h	Δ24-0
*Bifidobacterium*	C	7.29	7.41	0.12	5.31	7.10	1.79	8.20	8.21	0.01
	BC4	7.41	7.59	0.17	6.59	7.78	1.19	8.20	8.25	0.04
	BC9	7.41	7.40	−0.01	4.53	7.44	2.92	8.33	8.31	−0.01
	BC4 + BC9	7.49	7.48	−0.01	5.62	8.44	2.82	8.34	7.58	−0.76
	E-BC4	7.51	7.51	0.00	5.68	7.10	1.42	8.35	7.68	−0.67
	E-BC9	7.49	7.51	0.02	4.21	7.90	3.69	8.24	7.98	−0.26
	E-BC4 + BC9	7.57	7.34	−0.23	6.93	7.85	0.92	8.33	7.72	−0.61
*Lactobacillus*	C	4.27	4.36	0.09	b.d.l. ^1^	b.d.l.		4.20	4.19	−0.01
	BC4	4.28	5.70	1.42	b.d.l.	b.d.l.		6.04	5.84	−0.20
	BC9	4.80	4.59	−0.21	b.d.l.	b.d.l.		5.30	4.96	−0.34
	BC4 + BC9	5.82	5.33	−0.49	b.d.l.	4.88		6.13	5.30	−0.82
	E-BC4	6.46	5.09	−1.37	b.d.l.	b.d.l.		5.92	5.22	−0.70
	E-BC9	6.37	5.47	−0.90	b.d.l.	3.61		6.35	5.33	−1.01
	E-BC4 + BC9	5.82	5.40	−0.42	b.d.l.	b.d.l.		6.31	5.81	−0.50
*Enterobacteriaceae*	C	8.17	8.36	0.19	6.63	6.21	−0.42	8.38	7.77	−0.61
	BC4	8.25	8.45	0.20	6.87	7.41	0.54	8.29	7.40	−0.89
	BC9	8.16	8.15	−0.01	6.16	7.46	1.31	8.42	6.84	−1.58
	BC4 + BC9	8.27	8.24	−0.03	7.06	7.55	0.49	8.25	6.38	−1.86
	E-BC4	8.32	8.48	0.16	6.99	7.23	0.24	8.29	6.96	−1.33
	E-BC9	8.15	8.29	0.14	5.62	7.65	2.02	7.97	6.86	−1.12
	E-BC4 + BC9	8.40	8.14	−0.26	7.11	7.16	0.04	8.35	6.87	−1.48

^1^: b.d.l. means below the detection limit. The detection limit is 3.5 log DNA cells/g fecal culture.

## Data Availability

The original contributions presented in the study are included in the article, further inquiries can be directed to the corresponding authors.
